# Inflammatory profile of convalescent plasma to treat COVID: Impact of amotosalen/UVA pathogen reduction technology

**DOI:** 10.3389/fimmu.2022.1034379

**Published:** 2022-10-07

**Authors:** Fabrice Cognasse, Hind Hamzeh-Cognasse, Anne-Claire Duchez, Natalia Shurko, Marie-Ange Eyraud, Charles-Antoine Arthaud, Amélie Prier, Marco Heestermans, Olivier Hequet, Brigitte Bonneaudeau, Sandrine Rochette-Eribon, Françoise Teyssier, Valérie Barlet-Excoffier, Patricia Chavarin, Dominique Legrand, Pascale Richard, Pascal Morel, Nuala Mooney, Pierre Tiberghien

**Affiliations:** ^1^Etablissement Français du Sang Auvergne-Rhône-Alpes, Saint-Etienne, France; ^2^Université Jean Monnet, Mines Saint-Étienne, INSERM (Institut National de la Santé et de la Recherche Médicale), U 1059 Sainbiose, (SAnté INgéniérie BIOlogie St-Etienne), Saint-Étienne, France; ^3^Institute of Blood Pathology and Transfusion Medicine NAMS (National Academy of Medical Sciences) of Ukraine, Lviv, Ukraine; ^4^CIRI, International Center for Infectiology Research, INSERM (Institut National de la Santé et de la Recherche Médicale) U1111, Université de Lyon, Lyon, France; ^5^Etablissement Français du Sang, La Plaine St Denis, France; ^6^UMR (Unité mixte de recherche) RIGHT U1098, INSERM, Etablissement Français du Sang, Université de Franche-Comté, Besançon, France; ^7^Human Immunology, Pathophysiology and Immunotherapy, INSERM (Institut National de la Santé et de la Recherche Médicale) U976, Paris, France

**Keywords:** COVID-19, convalescent plasma, inflammation, cytokine, endothelial cell

## Abstract

Blood products in therapeutic transfusion are now commonly acknowledged to contain biologically active constituents during the processes of preparation. In the midst of a worldwide COVID-19 pandemic, preliminary evidence suggests that convalescent plasma may lessen the severity of COVID-19 if administered early in the disease, particularly in patients with profound B-cell lymphopenia and prolonged COVID-19 symptoms. This study examined the influence of photochemical Pathogen Reduction Treatment (PRT) using amotosalen‐HCl and UVA light in comparison with untreated control convalescent plasma (n= 72 – paired samples) - cFFP, regarding soluble inflammatory factors: sCD40L, IFN-alpha, IFN-beta, IFN-gamma, IL-1 beta, IL-6, IL-8, IL-10, IL-18, TNF-alpha and *ex-vivo* inflammatory bioactivity on endothelial cells. We didn’t observe significant modulation of the majority of inflammatory soluble factors (8 of 10 molecules tested) pre- or post-PRT. We noted that IL-8 concentrations were significantly decreased in cFFP with PRT, whereas the IL-18 concentration was increased by PRT. In contrast, endothelial cell release of IL-6 was similar whether cFFP was pre-treated with or without PRT. Expression of CD54 and CD31 in the presence of cFFP were similar to control levels, and both were significant decreased in when cFFP had been pre-treated by PRT. It will be interesting to continue investigations of IL-18 and IL-8, and the physiopathological effect of PRT- treated convalescent plasma and in clinical trials. But overall, it appears that cFFP post-PRT were not excessively pro-inflammatory. Further research, including a careful clinical evaluation of CCP-treated patients, will be required to thoroughly define the clinical relevance of these findings.

## Introduction

In order to improve the survival of COVID-19 patients with extreme acute respiratory syndromes of viral etiology, convalescent plasma therapy, *i.e.* passive polyclonal antibody administration to provide immediate immunity has been used (1). Patients who have recovered from COVID-19 and have a high plasma titer of neutralizing antibody may be a valuable source of convalescent plasma. Nevertheless, a therapeutic strategy in COVID-19 has not yet been tested regarding the balance between possible clinical advantages and the risks of convalescent blood product transfusion. Moreover, the potential association between the effectiveness of convalescent plasma and the inflammatory characteristics of this plasma has never been established, to our knowledge. Cytokines and/or chemokines are potent modulators of many immune response characteristics including inflammation. Such molecules are major inflammatory response effectors and regulators, which influence immune cell function (2). Inflammatory cytokines/chemokines also interfere with coagulation by fostering a pro-coagulant state that maintains inflammation (3). Plasma transfusion can modulate innate immune responses; however, the immunomodulatory capacity of various plasma products is little understood. Considering that most previous immune modulation research centered on red blood cell products or platelet concentrates linked to transfusion, even less is known about the immunomodulatory effects of plasma products (4, 5).

Although not completely understood, the pathological mechanisms underlying lung injury following the transfusion of Fresh Frozen Plasma (FFP) are thought to result from an inflammatory response involving neutrophil infiltration into the lungs and elevated interleukin (IL)-8 and IL-1 pulmonary levels, as observed in TRALI patients (6, 7). Several studies have shown that pathogen reduction technologies reduced the potential risk of transfusion-transmitted infections (8, 9), still, to our knowledge, convalescent plasma (cFFP) and the inflammatory properties of this plasma have not been investigated. Therefore, in the perspective of a therapeutic strategy in COVID-19, it is essential to characterize precisely the pro-inflammatory content and ability of plasmas that will be transfused to patients, in addition to determining the neutralizing antibody capacity of these plasmas.

## Material and methods

### Donor recruitment and sample collection

Convalescent patients eligible for plasma donation were asked to undergo plasma apheresis, as described recently (10). In France, plasma collection is recommended no earlier than 14 to 28 days after symptom resolution and plasma for transfusion currently undergoes pathogen reduction or quarantine. Once treated and qualified, plasma was cryopreserved (in 200–250 ml units) and made available for clinical use. Anti–SARS-CoV-2 antibody content was assessed in each donation, with a requirement for a SARS-CoV-2 seroneutralization titer of ≥ 40 and/or an immunoglobulin G (IgG) enzyme-linked immunosorbent assay (EUROIMMUN, Bussy-Saint-Martin, France) ratio > 5.6 (11). cFFP (n=72) units were produced in one regional center of the National Blood Service (EFS Auvergne-Rhône-Alpes). cFFP were collected from anonymous regular blood donors who had accepted, after receiving specific information, that their blood samples be used for research purposes and had signed a consent form approved by the regulatory authorities. Written informed consent was obtained from all the patients or their trusted persons. Data collection from the PLASMACOV cohort was approved by the French national ethics committee (2020-A00728-31) (12, 13). Apheresis was carried out using a plasma collection system (Auto-C or Aurora, Fresenius Kabi, BadHomburg, Germany) containing the anticoagulant Citrate Dextrose Solution (ACD, Macopharma, Tourcoing, France). After a leukofiltration step, a sample (2 mL) of each unit of convalescent plasma without pathogen reduction technology (PRT - INTERCEPT Blood System - Cerus Corp, Concord, CA) was aliquoted. A 2 mL sample of the same cFFP that met treatment criteria for amotosalen/UVA‐PRT was used for pathogen reduction according to the manufacturers instructions (14). Before and after PRT treatment, samples of each cFFP were stored at -80°C before use.

### Soluble inflammatory factor assays in cFFP

Levels of various soluble inflammatory factors (sCD40 Ligand, IFN-alpha, IFN-beta, IFN-gamma, IL-1 beta, IL-6, IL-8, IL-10, IL-18, and TNF-alpha) were quantified in cFFP with or without PRT using Luminex Technology, according to the manufacturer’s instructions (Bio-Techne, Minneapolis, US). Absorbance at 450 nm was determined using an enzyme-linked immunosorbent assay reader (Magellan Sunrise software, Tecan Group Ltd., Lyon, France).

### EA.hy926: Endothelial cell culture

EA.hy926 is a permanent cell line derived by fusing human umbilical vein endothelial cells (HUVEC) with the cell line A549 (15). The EA.hy926 cell line was obtained from the American Type Culture Collection (ATCC, Manassas, Virginia, USA). EA.hy926 was cultured as described (16) in 6-well and 96-well plates in order to obtain approximately 10^6^ cells/ml. The cell number was quantified with a TC10 Automated Cell Counter (Bio-Rad, Marnes-la-Coquette, France). Cells were grown until confluent then passaged with 0.25% trypsin (Sigma-Aldrich, Saint-Quentin-Fallavier, France).

### Stimulation of EA.hy926

EA.hy926 endothelial cells were cultured in 6- (1x10^6^ cells/well) or 96-well plates (5x10^4^cells/well) and grown for 48 hours to reach confluence prior to the experiment. Confluent endothelial monolayers were washed and incubated for 24 hours at 37°C and underwent the following treatments: control wells were incubated with DMEM only, test wells with convalescent FFP with or without PRT diluted at 1/5 in DMEM, and positive control wells were stimulated by recombinant human TNF-alpha (PeproTech, Neuilly-sur-Seine, France) at a final concentration of 100 pg/ml. After 24 hours of incubation, supernatants were recovered and frozen at -80°C. After washing in phosphate buffered saline (PBS), the cells were centrifuged for 5 minutes at 300 g and 22°C. The EA.hy926 Endothelial cells pellets were resuspended in 1% paraformaldehyde in PBS for 30 minutes and then washed. The fixed cells were resuspended in PBS for further analyses as described below.

### Flow cytometry analyses

The surface phenotype of EA.hy926 endothelial cells was determined by flow cytometry. Cells were fixed as described above. Direct labelling was performed for the following markers: endoglin/CD105 (BD Biosciences, Le Pont de Claix, France), ICAM-1/CD54 (BD Biosciences, Le Pont de Claix, France), and Platelet endothelial cell adhesion molecule PECAM1/CD31 (BD Biosciences, Le Pont de Claix, France). The cells were incubated for 30 minutes with antibodies. In all experiments, background labelling was assessed using the relevant fluorochrome-conjugated mouse IgG isotype control (BD Biosciences, Le Pont de Claix, France). Cells were centrifuged at 300 g for 5 minutes at 22°C then washed in PBS. Data acquisition was performed using a Guava easyCyte HT Flow Cytometer (Merck Millipore, Molsheim, France) and analysis carried out using the Incyte program. At least 10,000 events were collected for each sample.

### Endothelial cell IL-6 quantification

The production of soluble cytokines in culture supernatants of EA.hy926 Endothelial cells both stimulated and not stimulated by convalescent plasma or not was measured using the specific enzyme-linked immunosorbent assays (ELISA). Interleukin-6 levels (DuoSet) were measured by commercial ELISA kit (DuoSet - R&D Systems, Lille, France) according to the manufacturer’s instructions. Absorbance at 450 nm was determined with an ELISA reader (Magellan Sunrise software, Tecan Group Ltd., Lyon, France).

### Statistical analysis

The data was not normally distributed. The results were presented as scatter dot plots and red lines show the median of raw data. Statistical analysis was performed using paired t-tests (GraphPad™, La Jolla, CA). Statistically significant differences required a p value < 0.05 (*p<0.05, **p<0.01, ***p<0.001). Group comparisons were made using a one-way analysis of variance (ANOVA) followed by a Kruskal-Wallis test and a Dunn’s post-test. The two-tailed t-test and Mann-Whitney test were used to compare two groups (GraphPad Software, La Jolla, California, USA).

## Results and discussion

**Figure 1:**
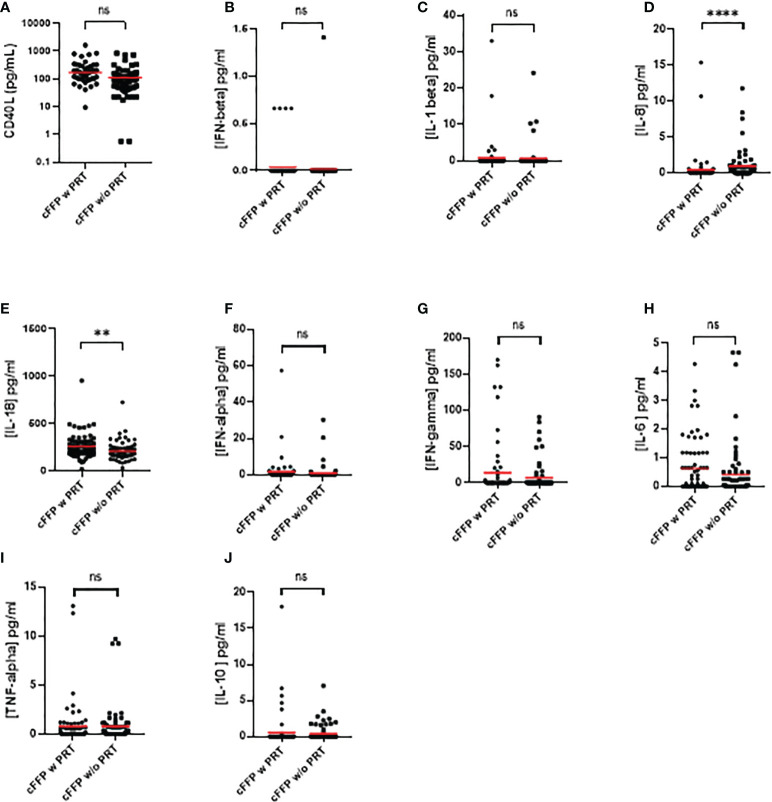
Quantification of soluble sCD40 Ligand **(A)**, IFN-alpha **(B)**, IFN-beta **(C)**, IFN-gamma **(D)**, IL-1 beta **(E)**, IL-6 **(F)**, IL-10 **(G)**, TNF-alpha **(H)**, IL-8 **(I**), and IL-18 **(J)** in convalescent FFP (cFFP) with or without PRT. The concentration of soluble inflammatory factors was quantified by Luminex technology. Values shown are deducted from background levels. Data (Scatter plot and mean; n = 72 for each group. Significance between samples was assessed using a paired sample. P values < 0.05 were considered to be significant (ns, not significant; ** < 0.01; **** < 0.0001).

Contents of sCD40 Ligand (A), IFN-alpha (B), IFN-beta (C), IFN-gamma (D), IL-1 beta (E), IL-6 (F), IL-10 (G) and TNF-alpha (H) in cFFP with PRT were not significantly different (P > 0.05) from untreated FFP (without PRT) (**Table 1**) - **(** **Figures 1A–H****)**, contrary to IL-8 (**Figure 1I**) and IL-18 (**Figure 1J**). Although the values are relatively low, we observed a significantly higher concentration of IL-8 in convalescent FFP without PRT compared to with PRT, respectively 0.9501 ± 2.012 vs. 0.4664 ± 2.203 pg/mL, this may indicate a less inflammatory plasma preparation after PRT treatment. In contrast, a significantly higher concentration of IL-18 is observed in convalescent FFP with PRT compared to without PRT; 258.1 ± 127.5 pg/mL vs. 207.6 ± 96.25 pg/mL respectively).

**Table 1:** Descriptive statistic of database.

		Number of values	Minimum (pg/mL)	25% Percentile (pg/mL)	Median (pg/mL)	75% Percentile (pg/mL)	Maximum (pg/mL)	Range (pg/mL)	Mean (pg/mL)	Std. Deviation	Std. Error of Mean	Lower 95% CI of mean	Upper 95% CI of mean	Coefficient of variation
**sCD40 Ligand**	cFFP w PRT	72	0	0	108.5	198.9	1655	1655	**172.3**	256.3	30.2	112.1	232.6	148.7%
	cFFP w/o PRT	72	0	18.65	67.57	126.2	848	848	**112.3**	165.5	19.51	73.36	151.2	147.4%
**IFN-alpha**	cFFP w PRT	72	0	0	0	0.91	57.41	57.41	**1.692**	7.23	0.852	-0.0066	3.391	427.2%
	cFFP w/o PRT	72	0	0	0	0	30.38	30.38	**0.985**	4.396	0.518	-0.0478	2.018	446.2%
**IFN-beta**	cFFP w PRT	72	0	0	0	0	0.66	0.66	**0.0366**	0.152	0.017	0.0008	0.0724	415.2%
	cFFP w/o PRT	72	0	0	0	0	1.41	1,41	**0,0195**	0,166	0,019	-0,0194	0,0586	848,5%
**IFN-gamma**	cFFP w PRT	72	0	0	0	0,83	169,9	169,9	**13,86**	38,02	4,48	4,924	22,79	274,3%
	cFFP w/o PRT	72	0	0	0	0	90,79	90,79	**7,057**	19,51	2,299	2,473	11,64	276,4%
**IL-1 beta**	cFFP w PRT	72	0	0	0	0	33	33	**0,857**	4,422	0,521	-0,1821	1,896	516,0%
	cFFP w/o PRT	72	0	0	0	0	24,21	24,21	**0,757**	3,435	0,404	-0,0494	1,565	453,3%
**IL-6**	cFFP w PRT	72	0	0	0,1	1,168	4,28	4,28	**0,627**	0,943	0,111	0,4063	0,8495	150,2%
	cFFP w/o PRT	72	0	0	0	0,26	4,68	4,68	**0,413**	0,973	0,114	0,1844	0,6417	235,6%
**IL-10**	cFFP w PRT	72	0	0	0	0	18,03	18,03	**0,567**	2,425	0,285	-0.0018	1.138	427.0%
	cFFP w/o PRT	72	0	0	0	0	7.05	7.05	**0.418**	1.114	0.131	0.1571	0.6804	265.9%
**TNF-alpha**	cFFP w PRT	72	0	0	0	0.68	13.08	13.08	**0.776**	2.181	0.257	0.2638	1.289	280.9%
	cFFP w/o PRT	72	0	0	0.18	0.73	9.71	9.71	**0.779**	1.895	0.223	0.3343	1.225	243.1%
**IL-8**	cFFP w PRT	72	0	0	0	0	15.4	15.4	**0.466**	2.203	0.259	-0.0512	0.9841	472.3%
	cFFP w/o PRT	72	0	0	0.32	0.79	11.8	11.8	**0.950**	2.012	0.237	0.4773	1.423	211.8%
**IL-18**	cFFP w PRT	72	18.51	185.2	223.8	306.3	953.9	935.4	**258.1**	127.5	15.03	228.1	288	49.40%
	cFFP w/o PRT	72	30.51	155.5	184.5	240.6	726	695.5	**207.6**	96.25	11.34	185	230.2	46.36%

The Mean (pg/mL), a more significant variable is represented by the bold values.

One limit of this study concerns the high cytokine profile heterogeneity. Interpretation of cytokine data in such studies is frequently made by comparing the mean levels in several experimental conditions. Our data show that the mean and median values are different. Outliers affect the mean value of the data but have little effect on the median or mode of a given set of data. Analysis of the outlier cFFPs with or without PRT could be interesting, particularly in a clinical context. Finally, clinical aspects were less detailed in our study but efficacy of Convalescent Plasma to Treat COVID-19 Patients, a Nested Trial in the CORIMUNO-19 Cohort (CORIPLASM) (NCT04345991) is under analysis.

**Figure 2:**
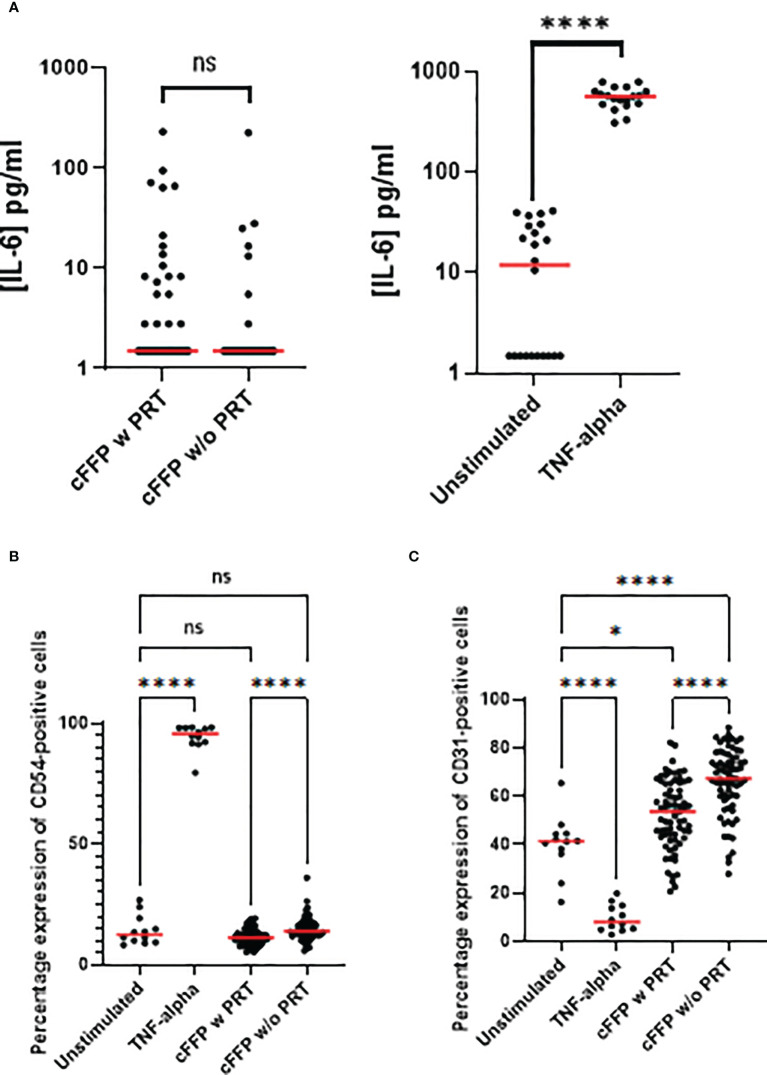
Endothelial cells (EA.hy926 cells) activation focusing on IL-6 release and on membrane expression. EA.hy926 cells were stimulated with convalescent FFP (cFFP) with (n = 72) - considered as the gold standard - or without PRT (n = 72). EA.hy926 cells were stimulated with negative and positive controls. TNF-α stimulation was considered the positive control (n = 18) and unstimulated EA.hy926 cells were viewed as the negative control (n = 19). Bioactivity in EA.hy926 endothelial cells was measured with IL-6 release **(A)**, with or without stimulation. Bioactivity in EA.hy926 endothelial cells was measured with FCM focusing on membrane expression of CD54 **(B)** and CD31 **(C)** with or without stimulation. Scatter dot plots represent the data, and the red line denotes the median of raw data. Statistical analyses were performed using one-way Anova with multiple comparisons (P-values<0.05 were considered significant; * < 0.05; **** < 0.0001).

Production of IL-6 by endothelial cells has been widely documented in pro-inflammatory environments. We therefore examined Il-6 release after endothelial cell activation with cFFP. We did not observe any PRT-related modulation of IL-6 release (n=72), in contrast to IL-6 levels activated by TNF-α stimulation (n=12) **(** **Figure 2A****).**

To further evaluate the effect of cFFP with or without PRT treatment (n=72) on endothelial cells, plasma was added to EA.hy926 cell cultures and membrane expression of adhesion molecules was then evaluated. The percentage expression of CD105 (data not shown) was unchanged in the presence of cFFP with or without PRT treatment or by recombinant human TNF-α at a final concentration of 100 pg/ml (n=12).

CD54 **(** **Figure 2B****)** and CD31 **(** **Figure 2C****)** two crucial endothelial cell adhesion molecules, that are implicated in endothelial permeability, and transendothelial leukocyte migration had similar expression levels as in controls. We did not observe significant modulation of CD54 in the presence of cFFP with or without PRT treatment compared to unstimulated EA.hy926 cells. However, expression of CD31 was significantly increased in the presence of cFFP with or without PRT treatment compared to unstimulated EA.hy926 cells, respectively p=0.207 and p<0.0001 **(** **Figure 2C****).** Furthermore, both CD54 and CD31 expression were significantly decreased by exposure to cFFP that had undergone PRT treatment **(** **Figure 2C****).** As expected, TNF-α stimulation reduced the expression of PECAM-1 (CD31). This molecule is highly implicated in the process of trans-endothelial-migration [reviewed in (17) and (18)]. Moreover, as previously reported, TNF-α stimulation increased the expression of ICAM (CD54) (19) (**Figures 2B, C**). Biological response modifiers (BRMs), such as anti-microparticles, lipids, and cytokines/chemokines, and particularly sCD40L, have been linked to inflammatory Serious Adverse Reactions (SARs), such as Transfusion-related acute lung injury (TRALI). sCD40L is a platelet-derived proinflammatory mediator that accumulates during platelet concentrate or FFP storage. Because COVID-19 patients are more likely to develop Acute Respiratory Distress Syndrome (ARDS), the absence of significant modulation of sCD40L in cFFP with PRT was reassuring.

Treatment of cFFP by PRT clearly increased levels of the pro-inflammatory cytokine IL-18. Serum IL-18 levels in healthy subjects ranged from 80 to 120 pg/mL (20). An increase of IL-18 concentration has been observed in the serum of i) adult and pediatric patients with Crohn’s disease (21) (in the order of 400 pg/mL), ii) patients with coronary artery disease (22, 23) (in the range of 70 to 300 pg/mL) an iii) patients with ARDS (in the order of 600 pg/mL), and correlated with severity score and death (24). In the light of these studies, clinical research will be performed to evaluate the transfusion efficacy related to the transfusion of convalescent or non-convalescent plasma as a therapeutic support in ARDS patients.

In the context of COVID-19, Zachary B Zalinger et al. suggested that inflammasome signaling is largely protective in murine coronavirus infection, largely due to the pro-inflammatory effects of IL-18 (25). However, another report noted significantly elevated levels of IL-18 in the plasma of COVID-19 patients (26). The levels of other inflammatory cytokines/chemokines IL-1b, IL-1RA, IL-6, IL-8, IL-18 and TNFα were not higher in critically ill patients with COVID-19 than in critically ill patients admitted for ARDS or sepsis (27). Finally, although high concentrations of cytokines/chemokines have been widely described in COVID-19 patients, the vast majority (including IL-6, IL-10, IL-18, CTACK and IFN-γ) do not appear to be prognostic, as they do not always differentiate between moderate and severe cases (28). In a model of the immune cell interaction between DC and B cells in COVID-19 patients, IL-18 was found to be critical for antibody production by B cells, suggesting its importance in recovery (29). It will be interesting to see whether IL-18 present in convalescent plasma correlates with plasma titers of neutralizing antibody.

Convalescent plasma from recovered COVID-19 patients contain neutralizing antibodies against the spike protein of SARS CoV-2, which may benefit severely sick COVID-19 patients by neutralizing the virus and halting its replication in the host. Early administration of high-titer convalescent plasma against SARS-CoV-2 to mildly ill infected older adults reduced the progression of Covid-19. The use of inflammatory convalescent plasma to treat COVID-19 may be an interesting approach that has not yet been fully studied and may potentially increase the efficacy of transfusion.

The concentration of IL-8 in convalescent plasma with or without PRT treatment was low, with a median of 0 [0-15.40] and 0.32 [0-11.80] pg/mL respectively. In healthy subjects, the median serum IL-8 level was 87.45 pg/mL (5–7500), which exceeded the normal range (< 62 pg/mL) and indicated an increase in serum IL-8 level.

Anil Bagri et al. (30) measured the level of cytokines in CCP and the impact of Amotosalen/UVA pathogen reduction treatment (A/UVA-PRT), and compared Pre-PRT and Post-PRT levels using a highly sensitive and specific Luminex-based multiplexed cytokine panel. Concerning the common soluble inflammatory factors, TNF alpha, IL-6, IL-10, we did not observe significant modulation pre-PRT or post-PRT. Overall, Anil Bagri et al. (30) did not observe significant modulation of cytokines studied (in agreement with our study) including GM-CSF, Interleukin (IL)-2, IL-3, IL-4, IL-5, IL-7 and IL-17. We have reported similar results concerning sCD40 Ligand, IFN-alpha, IFN-beta, IFN-gamma, IL-1 beta, IL-6, IL-10 and TNF-alpha in cFFP with or without PRT. However, Anil Bagri et al. reported that PRT reduced the levels of all three pro-inflammatory cytokines (MIP-1β p, MCP-1 and IL-1β p), this is in keeping with our results concerning IL-8 (30).

There are several potential explanations for the higher IL-18 concentration detected in PRT cFFP i) the soluble IL-18Rα complex is composed of the soluble forms of the IL-18Rα and IL-18Rβ chains and binds IL-18. We can’t exclude that PRT cFFP could decrease the cFFP concentration of the soluble IL-18R resulting in increased detection of free IL-18; ii) The sequestration of IL-18 by its soluble decoy receptor IL-18-Binding Protein (IL-18BP) is critical to the regulation of IL-18 activity and a similar mechanism could result in a decreased concentration of IL-18-BP in PRT cFFP leading to detection of higher levels of free IL-18. Finally, upon exposure to UV radiation, both precursors (pro-IL-18) are cleaved by caspase-1. Caspase-1plays a major role in the cleavage of the IL-18 precursor, and we cannot exclude that PRT does not increase caspase-1 activity resulting in a higher IL-18 concentration in PRT cFFP.

Cytokines/chemokines have been considered individually and we can’t exclude synergistic, antagonistic and additive effects also, the modulation of cytokine/chemokine concentration between the two conditions was noted but this was not reflected in their bioactivity on endothelial cells. IL-18 concentration was significantly elevated in cFFP PRT compared to cFFP w/o PRT, but this concentration is very low compared to that in serum samples from patients with COVID-19. Hospitalized. COVID-19 patients had higher IL-18 levels compared to healthy subjects (103 pg/mL vs. 310 pg/mL). Moreover, in the same report, the authors demonstrated that serum IL-18 concentrations are remarkably increased in patients with COVID-19 and correlated with other inflammatory markers and disease severity (31).

Moreover, this study examined whether convalescent plasma with or without PRT treatment modified the endothelial bioactivity. Concerning the EA.hy926 cell line, we did not observe any significant modulation of IL-6 release after activation with convalescent plasma with or without PRT treatment. Although CD54 expression in the presence of cFFP (with or without PRT treatment) remained close to control levels, expression was significantly decreased in the presence of cFFP pre-treated by PRT treatment. In contrast, CD31 expression was higher in endothelial cells treated by cFFP, importantly PRT treatment decreased the CD31 expression. Modulation of CD54 and/or CD31 may have important consequences because both CD54 have well-documented roles in the process of trans-endothelial-migration. This process has been intensely studied. Endothelial cell membrane CD54 has a role early in the transendothelial migration (TEM) of polymorphonuclear neutrophils and accumulates below the leukocyte in response to interactions with LFA-1 on the leukocyte. As TEM begins, ICAM-1 surrounds the leukocyte as it transmigrates. CD31 localizes both in the endothelial border and in Lateral Border recycling compartments LBRC and in the steady state 30% of total PECAM-1 is estimated to be within the LBRC. Endothelial CD31 is implicated in two critical and related signaling events in transmigration, by engaging in homotypic interactions with the leukocyte followed by a transient intracellular Ca^++^ increase. Disruption of CD31-CD31 homophilic interactions blocks both TEM and recycling of the LBRC and limitation of either of signaling event restricts TEM of neutrophils and/or monocytes. Therefore, the modification of CD54 and/or CD31 expression by PRT is likely to decrease both early and late stages of leukocyte binding and migration across the endothelial membrane and may thereby reduce inflammation.

Because some debate persists on the broad implementation of PRT regardless of consensus, it is crucial to continue investigations on both the physiopathology of intercept treated-blood products and post marketing clinical trials.

## Data availability statement

The raw data supporting the conclusions of this article will be made available by the authors, without undue reservation.

## Ethics statement

Data collection from the PLASMACOV cohort was approved by the French national ethics committee (2020-A00728-31). The patients/participants provided their written informed consent to participate in this study.

## Author contributions

CF and HH-C made the study hypothesis, wrote the manuscript, designed the protocol and trained personnel in blood banks. PM, NM, NS, and PT contributed to the writing of the manuscript. FC, HH-C, A-CD, NS, M-AE, C-AA, AP, MH, OH, BB, SR-E, FT, VB-E, PC, DL, PR, NM and PT collected samples, did the experiments and statistical analyses, and co-wrote the manuscript. All authors contributed to the article and approved the submitted version.

## Funding

This work was supported by grants from the French National Blood Service – EFS (Grant APR), France and the Association “Les Amis de Rémi” Savigneux, France.

## Acknowledgments

We would like to thank the medical staff and personnel of the Etablissement Français du Sang Auvergne-Rhone-Alpes, Saint-Etienne, France for technical support throughout our studies. We thank the blood donors for taking part in this study.

## Conflict of interest

The authors declare that the research was conducted in the absence of any commercial or financial relationships that could be construed as a potential conflict of interest.

## Publisher’s note

All claims expressed in this article are solely those of the authors and do not necessarily represent those of their affiliated organizations, or those of the publisher, the editors and the reviewers. Any product that may be evaluated in this article, or claim that may be made by its manufacturer, is not guaranteed or endorsed by the publisher.
